# CRHR1 Gene Copy Number Variations, Chronic Viral Infections, and Age as Correlates of Cognitive Impairment in Adults After SARS-CoV-2 Infection

**DOI:** 10.3390/cimb48010069

**Published:** 2026-01-08

**Authors:** Yekaterina Hovhannisyan, Hermine Yeritsyan, Hayk Harutyunyan, Allen Azizian, Konstantin Yenkoyan

**Affiliations:** 1 Neuroscience Laboratory, COBRAIN Center, Yerevan State Medical University Named After Mkhitar Heratsi, Yerevan 0025, Armenia; katyaarmdoc@yahoo.com (Y.H.); yeritsyan_hermine@yahoo.com (H.Y.); hayk@web.am (H.H.); 2 Neurology Service at Neurosurgery Comprehensive Stroke Center, Heratsi N1 University Clinic, Yerevan State Medical University Named After Mkhitar Heratsi, Yerevan 0025, Armenia; 3 Department of Criminology, California State University, Fresno, CA 93740, USA; aazizian@mail.fresnostate.edu; 4 Department of Medical Psychology, Yerevan State Medical University Named After Mkhitar Heratsi, Yerevan 0025, Armenia

**Keywords:** SARS-CoV-2 infection, copy number variations, *CRHR1* gene, cognitive impairment, young and middle-aged individuals, MOCA, RBANS

## Abstract

Cognitive impairment is a frequent but heterogeneous consequence of SARS-CoV-2 infection, with objective cognitive deficits not always aligning with subjective cognitive complaints. Age, nutritional status, and stress-related biological pathways may contribute to this variability. The corticotropin-releasing hormone receptor 1 (CRHR1), a key regulator of stress and neuroendocrine responses, represents a biologically plausible candidate for post-infection cognitive vulnerability. In this pilot case–control study, we investigated associations between *CRHR1* copy number variations (CNVs), prior viral exposures, and cognitive outcomes in adults following SARS-CoV-2 infection. Objective cognitive performance was assessed using the Montreal Cognitive Assessment (MoCA) and RBANS, alongside evaluation of subjective cognitive complaints and depressive symptoms. Analyses accounted for age and circulating levels of vitamins B12, B9, and vitamin D. *CRHR1* CNVs affecting specific exons (Exon 1 [210 nucleotides] and Exon 11) were associated with objective cognitive impairment, whereas subjective cognitive complaints were more closely related to depressive symptoms than measurable cognitive deficits. Associations with age and certain viral seropositivities (HSV-1, HSV-2, and Hepatitis A) were also observed with objective cognitive outcomes; however, these findings should be interpreted cautiously given their exploratory nature. This study highlights *CRHR1* CNVs as potential modifiers of objectively measured post-COVID-19 cognitive impairment and underscores the importance of distinguishing subjective cognitive complaints from objective cognitive dysfunction, providing a framework for future mechanistic and longitudinal studies.

## 1. Introduction

The period from December 2019 to May 2023 was marked by the global coronavirus pandemic caused by the SARS-CoV-2 virus [1]. However, large outbreaks of coronavirus infections had also occurred previously, including SARS-CoV in 2002 and MERS-CoV in 2012, both characterized by acute respiratory syndrome and, in some cases, death [2]. Coronavirus infections have been associated with neurological symptoms during both the acute and late stages, including memory loss, executive dysfunction, and depressive symptoms [3]. Memory impairment was reported in approximately one in five individuals infected with MERS-CoV, while executive function deficits were observed in one in three patients discharged after COVID-19 infection [4]. During the acute phase of COVID-19, up to 36% of patients and as many as 84% of hospitalized individuals experienced at least mild cognitive impairment [5], with 15% of outpatients and 34% of inpatients reporting difficulties with concentration and memory [6]. These observations highlight the neurological impact of coronavirus infections and underscore the importance of investigating factors that may contribute to cognitive decline in affected individuals.

Mood-related symptoms, such as feelings of depression, were also common among COVID-19 survivors, affecting an estimated 30–40% of individuals according to findings from a systematic review [7]. These conditions have a significant impact on cognitive function and quality of life [8], particularly in areas such as attention, concentration, memory, and speed in processing of information [9]. Approximately 40% of hospitalized patients with COVID-19 experienced some form of psychiatric symptoms [10]. It is noteworthy that depressive symptoms during the acute phase of infection are a stronger predictor of cognitive impairment than the duration of hospitalization, treatment in the intensive care unit, comorbidities, or other factors [9]. Psychiatric comorbidity was also observed in non-hospitalized patients, with 52% meeting the criteria for depression and anxiety [11].

According to a meta-analysis, anxiety (19%), depression (12.9%), and cognitive changes (20%) are among the most common symptoms even after the acute phase of COVID-19 [12]. However, cognitive impairment in individuals with clinically mild COVID-19 does not appear to result solely from mood disturbances, but rather, arises from complex, multifactorial causes that interact with other demographic and clinical characteristics [13]. Notably, symptoms such as “memory decline,” “anxiety,” and “depression” are more prominent in young and middle-aged individuals without clinical cognitive impairment [14,15,16]. Even among medical staff who worked with COVID-19 patients, greater epigenetic activation of certain stress-related genes was observed compared to medical staff who worked with other patients [17]. Whether these symptoms have a subjective or objective basis, they have become the focus of numerous studies. It has been shown that subjective symptoms in COVID-19 patients are primarily linked to anxiety and depression [18], and depression can mimic dementia and may exacerbate cognitive impairment in patients with dementia or mild cognitive impairment (MCI) [19].

These individual differences in memory and mood changes—often observed among younger populations after coronavirus infection—highlight the need to explore potential genetic predispositions that may influence these symptoms. In our previous study, we identified associations between cognitive impairment and copy number variations (CNVs) in genes such as *PSEN1* (exons 1, 9, and 12), *GRN* (exons 1, 6, and 12), and *MAPT* (exons 2 and 8), suggesting a genetic component to post-COVID-19 cognitive symptoms in young and middle-aged individuals [20]. Building on these findings, we sought to further investigate the relationship between genetic factors and neuropsychiatric symptoms, with a particular focus on the corticotropin-releasing hormone receptor 1 (*CRHR1*) gene.

Given its high expression in the central nervous system and its known role in mediating stress responses, *CRHR1* is a compelling candidate gene in the context of stress-related cognitive and mood disturbances. Although numerous studies have examined single-nucleotide polymorphisms (SNPs) in stress-related genes, copy number variations (CNVs) may produce more substantial functional consequences by altering gene dosage or disrupting exon-level gene structure. CNVs affecting coding or regulatory regions can directly influence transcription, alternative splicing, and receptor expression, potentially exerting stronger biological effects than single-nucleotide changes [21]. CNVs affecting neuronal and synaptic genes have been associated with a range of cognitive and neurodevelopmental disorders, including intellectual disability, schizophrenia, and Alzheimer’s disease [22]. Given the dosage-sensitive nature of *CRHR1* expression, we focused our analysis on CNVs rather than SNPs to capture structural changes with potentially greater physiological impact․ In this study, we examined whether CNVs in the *CRHR1* gene may underlie the depressive symptoms and cognitive impairment observed following SARS-CoV-2 infection.

In response to stress, corticotropin-releasing hormone (CRH), a key-player, participates in neuroendocrine, autonomic, and behavioral changes [23]. Stress stimulates the production of CRH in the paraventricular nuclei of the hypothalamus, which binds to CRHR-1 receptors in the anterior pituitary, leading to the production of adrenocorticotropic hormone (ACTH). ACTH, in turn, stimulates cortisol production in the adrenal cortex, helping the body adapt to stress [24]. Depending on its location, CRHR1 may play a dual role (anxiolytic or anxiogenic) in the development of anxiety through glutamatergic or dopaminergic antagonistic activation, balancing the emotional response to stress [25].

The body’s adaptation to stress is regulated by the hypothalamic–pituitary–adrenal (HPA) axis. Several mutations in genes controlling the HPA axis—CRH, corticotropin-releasing hormone-binding protein (CRHBP), *CRHR1* and CRHR2, glucocorticoid receptor (NR3C1), mineralocorticoid receptor (NR3C2), and FK506-binding protein 5 (FKBP5)—also play important roles in the development of cognitive dysfunction and mental illness [26].

The *CRHR1* gene encodes the G protein-coupled CRHR1 receptor, which binds CRH family neuropeptides and activates the signaling pathways responsible for a range of physiological processes, including stress, reproduction, immune response, and obesity. Located on chromosome 17q21.31, the gene comprises 14 exons, and alternative splicing generates multiple transcript variants [27]. The biological activity of CRH is mediated by two receptor types—CRHR1 and CRHR2—each encoded by distinct genes but sharing approximately 70% amino acid identity [28]. Their functional divergence is driven largely by differences in their ligand-binding domains, which result in distinct physiological roles [29].

CRHR1 is widely distributed throughout the central nervous system (CNS), including the cortex, cerebellum, hippocampus, amygdala, olfactory bulb, basal ganglia, thalamus, and paraventricular gray matter. In contrast, CRHR2 is primarily found in subcortical structures such as the lateral septum, striatum, ventral hypothalamic nucleus, and amygdala [30,31]. CRHR1 plays a critical role in the acute phase of the stress-induced HPA response, while CRHR2 is involved in the recovery phase [23].

Dysregulation of CRHR1 and CRH is causally associated with stress-related pathologies, including mood and anxiety disorders. Elevated CRH levels have been observed in the cerebrospinal fluid of depressed patients, along with decreased CRH binding in the frontal cortex [32]. Limbic CRHR1 is essential for HPA axis feedback in response to stress, but recent findings suggest that it also modulates anxiety-related behavior independently of the HPA axis [33]. While CRHR1 deficiency in the forebrain reduces stress-induced cognitive impairment [34], certain mutations, such as the *CRHR1* TAT haplotype, are linked to cognitive dysfunction, including difficulties in decision-making, reasoning, learning, and memory in the context of depression [35]. Some mutations do not decrease CRHR1′s affinity for CRH but increase it. For example, the *CRHR1* p.V97M mutation in dogs increases binding between CRHR1 and CRH by 17%, which may cause conditions like pituitary hyperadrenocorticism [36].

Risk factors for the development of depression include hypodynamia, alcoholism, cardiovascular diseases, tumors, diabetes, respiratory diseases [37], and specific genetic predispositions [38]. Beyond SARS-CoV-2 infection and depression, cognitive impairment may also be influenced by other risk factors, including herpes simplex virus types 1 and 2 (HSV-1, HSV-2), cytomegalovirus (CMV), Epstein–Barr virus (EBV) [39], Hepatitis A [40], Hepatitis B [41], Hepatitis C [42], HIV [43], and hypovitaminosis D [44], B12 [45], and B9 [46].

This study aimed to investigate the role of *CRHR1* gene CNVs in objectively measured cognitive impairment and self-reported cognitive decline following SARS-CoV-2 infection. Additionally, the study explored potential risk factors for cognitive decline, including age, chronic viral infections (such as HSV-1, HSV-2, and Hepatitis A), depression, gender, and vitamin deficiencies. By examining both genetic predispositions and environmental risk factors, this research seeks to uncover new insights into the complex interplay between biological and external influences on objective and subjective cognitive decline in SARS-CoV-2-infected adults. The study was designed as a pilot to evaluate the feasibility and potential effects of the examined markers in a real-world setting. Due to its exploratory nature, the sample size is limited, and all eligible and available participants during the study period were included. While the small sample size restricts the generalizability of the findings, the results may guide the design of a larger, adequately powered future study. Because all participants in this study had confirmed SARS-CoV-2 infection, the aim of the study was not to determine whether SARS-CoV-2 itself affects *CRHR1* CNVs. Rather, we investigated whether inter-individual variation in *CRHR1* CNVs is associated with cognitive outcomes in the post-COVID-19 period.

## 2. Materials and Methods

### 2.1. Study Design

This study was designed as a case–control investigation to explore the genetic factors associated with cognitive impairment in survivors of SARS-CoV-2.

**Figure 1:**
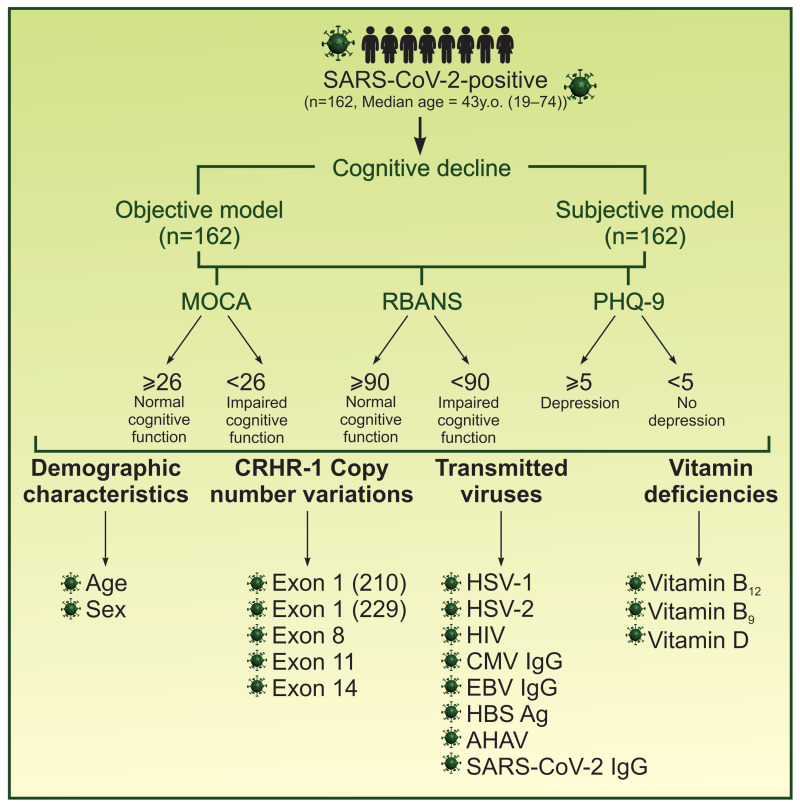
Graphical illustration of study design. SARS-CoV-2-infected adults (n = 162) were analyzed using two complementary approaches: the objective model, assessing cognitive impairment with MOCA and RBANS, and the subjective model, based on self-reported cognitive decline. All participants underwent testing for *CRHR1* CNVs, depression (PHQ-9), and additional risk factors, including age; sex; vitamin D, B12, and B9 deficiencies; and prior viral infections (HSV-1, HSV-2, CMV, EBV, Hepatitis A–C, HIV). Both models included the same cohort, allowing parallel analysis of objective and subjective cognitive outcomes.

Initially, two research models were selected due to the absence of prior genetic research on cognitive impairment in our population: in the first model, we aimed to identify the presence of the *CRHR1* gene CNVs among our population, whereas the second model evaluated the association of subjective cognitive impairment between genetic variations, depression, and other risk factors (Figure 1).

In the first model, patients were categorized into two groups based on the objectivity of cognitive impairment (MOCA, RBANS) and subsequently compared regarding *CRHR1* gene CNVs, depressive symptoms (PHQ-9), and other risk factors (age; sex; hypovitaminosis D, B12, and B9; and viral infections (SARS-CoV-2; HSV-1; HSV-2; CMV; EBV; Hepatitis A, B, and C; and HIV)). The case group consisted of individuals with objective cognitive impairment, while the control group comprised individuals without objective cognitive impairment.

In the second model, patients were divided into two groups based on self-reported cognitive impairment. These groups were then compared in terms of genetic variations, depression, age, sex, vitamin deficiencies, and viral infections. The case group included individuals with self-reported cognitive impairment, while the control group consisted of individuals without self-reported cognitive impairment.

Self-reported cognitive decline was assessed using a questionnaire developed by our research group, consisting of 15 items evaluating post-COVID-19 cognitive functions, including memory, attention, and executive function. Participants responded with a “yes/no” answer, indicating whether they experienced difficulties in these areas after COVID-19. This assessment helped classify individuals into case and control groups.

### 2.2. Participants

A total of 162 participants (35% male, 65% female), aged 19 to 74 years (median age: 43) who had recovered from SARS-CoV-2 infection were included in the study. Participants were recruited from the Heratsi Hospital Complex in Yerevan, Armenia, between January 2020 and August 2022. Data was collected during either hospitalization or outpatient visits, through a single physical examination and a series of laboratory and functional tests. All participants provided informed consent and were of Armenian nationality. The inclusion criteria were as follows: age ≥ 18 years; confirmed SARS-CoV-2 infection via RT-PCR or rapid antigen test; non-impaired level of consciousness and no mechanical ventilation requirement, no prior cognitive impairment or severe organ failure.

### 2.3. Cognitive Assessments

Cognitive assessment was conducted using the Montreal Cognitive Assessment (MOCA) [47] and the Repeatable Battery for the Assessment of Neuropsychological Status (RBANS) [48]. MOCA was used to assess cognitive domains such as memory, attention, language, and executive functions. For the MOCA test, a cut-off score of 26/30 was chosen (a score of ≥26/30 was considered indicative of normal cognitive function, while a score of <26/30 indicated impaired cognitive function) [47].

RBANS (License Contract No. LSR-444967) was used to assess immediate memory, visuospatial construction, language, attention, and delayed memory. For the RBANS test, a threshold of 90 points was chosen, which varies by ±10 CD compared to the American standards (±10–15 CD is allowed according to the test guidelines) [48] and is more applicable to the Armenian population according to the average scores in this population and the results of a previous study [49].

A total of 162 adults who had recovered from SARS-CoV-2 infection completed the RBANS assessment, while 161 participants completed the MOCA; one participant did not complete the MOCA.

### 2.4. Depression Assessment

Depression was assessed using the Patient Health Questionnaire (PHQ-9). A score of ≥5 was indicative of the presence of depression [50].

Our objective in including the PHQ-9 was to screen depressive mood and examine its relationship with other variables, rather than to diagnose depression or its stages. The significant role of depression in subjective cognitive decline, particularly following COVID-19, has been well-established in the literature. While we recognize that PHQ-9 is a self-report measure, it is widely used in clinical and research settings due to its high specificity (88%) and sensitivity (88%) for major depression when a score of ≥10 is used.

### 2.5. Genetic Analysis

CNVs in the *CRHR1* gene were analyzed using the Multiplex Ligation-dependent Probe Amplification (MLPA) technique by employing a SALSA MLPA Probemix P275 MAPT-GRN (MRC-Holland, Amsterdam, The Netherlands) [51], a kit for assessing disorders that includes probes targeting 5 exons of the *CRHR1* gene, along with reference probes located on multiple autosomal chromosomes. The analyzed *CRHR1* exons were selected based on the design of this commercially available MLPA kit, which targets specific coding exons across the gene. In addition to technical coverage, the selected exons include regions encoding functionally important domains of the *CRHR1* receptor, including ligand-binding and intracellular signaling regions.

Specific exons analyzed included Exon 1 (length—210 nucleotides; SALSA MLPA probe—08366-L08220), Exon 1 (length—229 nucleotides; SALSA MLPA probe—08367-L08221), Exon 8 (length—475 nucleotides; SALSA MLPA probe—07859-L07620), Exon 11 (length—154 nucleotides; SALSA MLPA probe—08369-L08223), and Exon 14 (length—427 nucleotides; SALSA MLPA probe—08370-L08224). It has to be mentioned that Exon 1 (210) and Exon 1 (229) represent two independent MLPA probes targeting separate regions within Exon 1 of the *CRHR1* gene. Fragment analysis was carried out on a SeqStudio Genetic Analyzer (Thermo Fisher Scientific, Waltham, MA, USA), and data was analyzed using GeneMapper Software 5 (Thermo Fisher Scientific, Waltham, MA, USA).

CNVs were identified as either homozygous or heterozygous deletions or duplications in the analyzed gene. Genetic analysis focused on the presence of CNVs in the *CRHR1* gene, which was represented using an integer value: 0 for homozygous mutations, 1 for heterozygous mutations, and 2 for no mutations. However, in our current study, we identified only deletions.

### 2.6. Laboratory Measurements

Using the Roche Cobas e411 immunoassay system, tests for the following were conducted: Herpes simplex virus 1 (HSV-1) IgG antibodies (Elecsys HSV-1 IgG); Herpes simplex virus 2 (HSV-2) IgG antibodies (Elecsys HSV-2 IgG); Cytomegalovirus (CMV) IgG antibodies (Elecsys CMV IgG); Epstein–Barr virus (EBV) nuclear antigen-1 IgG antibodies (Elecsys EBV EBNA IgG); Hepatitis A IgG/IgM antibodies (Elecsys Anti-HAV II); Hepatitis B IgG/IgM antibodies (Elecsys Anti-HBV II); Hepatitis C IgG/IgM antibodies (Elecsys Anti-HCV II); HIV (Elecsys HIV combi PT); SARS-CoV-2 IgG/IgM antibodies (nucleocapsid, Elecsys Anti-SARS-CoV-2); Vitamin B12 (Elecsys Vitamin B12 II); Folate (Elecsys Folate III); and Vitamin D (Elecsys Vitamin D). Tests were properly calibrated and regularly controlled according to the manufacturer’s recommendations (Roche Diagnostics GmbH, Mannheim, Germany). SARS-CoV-2 exposure status was measured by RT-PCR and/or a rapid antigen test [52].

### 2.7. Statistical Methods

Descriptive statistics were first calculated, where medians and interquartile ranges were determined for continuous variables, while frequencies and percentages were reported for categorical variables. For comparative analysis, Fisher’s Exact Test was employed to compare categorical variables between groups. The Mann–Whitney–Wilcoxon Test was used for continuous variables to assess differences between groups.

To evaluate the relationship between different factors and the presence of cognitive impairment, regression models were used. Logistic Regression was applied for binary outcomes, such as the subjective presence of impairments, while Linear Regression was used for continuous outcomes, such as the MOCA score. These models aimed to estimate the association between cognitive impairment and several factors, including *CRHR1* CNVs, depression, viral infections, hypovitaminosis, and age. Independent variables included *CRHR1* CNV status; age; sex; depression (assessed by PHQ-9); HSV-1, HSV-2, and Hepatitis A seropositivity; and vitamin D, B9, and B12 deficiency. Both univariate and multivariate models were applied to control for potential confounding effects, with variables reaching *p* < 0.05 in the univariate analysis included in the multivariate model.

Statistical analyses were conducted using R version 4.4. All tests were two-sided, and a *p* value < 0.05 was considered statistically significant.

Analyses were performed using complete-case data. No imputation procedures were applied for missing values, and participants with missing data for a given analysis were excluded from that specific model. Given the exploratory, pilot nature of the study, no formal correction for multiple testing (e.g., Bonferroni or false discovery rate adjustment) was applied; therefore, the results should be interpreted as hypothesis-generating.

## 3. Results

### 3.1. CRHR1 Gene CNVs and Cognitive Impairment

**Figure 2:**
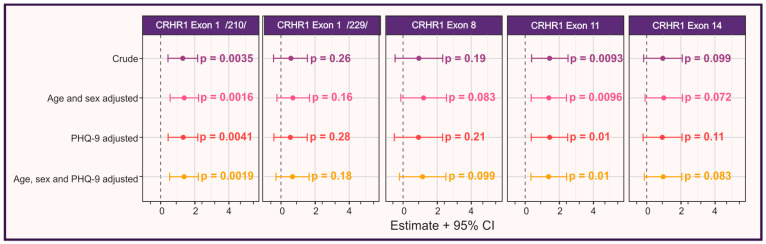
*CRHR1* gene copy number variations among patients with objective cognitive decline in univariate analysis and after adjustment for depression (assessed by the Patient Health Questionnaire-9 [PHQ-9]), age, and sex. Error bars represent 95% confidence intervals.

CNVs in the *CRHR1* gene were examined to assess their potential relationship with objective and subjective cognitive impairment. All detected CNVs represented exon deletions, with particular focus on Exons 1 (length variants 210 and 229), 8, 11, and 14. In the studied population, Exon 1 (210) CNVs were absent in 6% of participants, present in heterozygous form in 52%, and homozygous in 42% (Table 1 and Table 2). Participants carrying Exon 1 (210) deletions demonstrated significantly higher rates of objective cognitive impairment (*p* = 0.0035 for the crude analysis; *p* ≤ 0.0041 for adjusted models) (Figure 2). In contrast, the Exon 1 (229) deletion was predominantly homozygous (79% of participants; Table 1 and Table 2) and showed no significant association with cognitive decline in the univariate analyses (*p* > 0.16 for all models) (Figure 2).

The Exon 8 CNVs showed a different pattern: 17% of participants had no mutation, while 83% were heterozygous, with no homozygous individuals (Table 1 and Table 2). Although there was no clear trend for homozygosity, heterozygous carriers displayed a significant trend toward cognitive impairment according to the MOCA (*p* = 0.003 for proportions comparison between groups), but for proportions analyzed based on RBANS and Linear Regression analysis, it did not reach statistical significance (*p* > 0.1) (Table 1 and Figure 2). This indicates uncertainty of the signal.

The Exon 11 CNVs also revealed a predominant heterozygous state (64%) in participants, with 36% mutation-free (Table 1 and Table 2). A significant association between the heterozygous form of this mutation and cognitive decline was observed by both neurocognitive assessment tests and exists in regression analysis (*p* < 0.01 in all considered models) (Table 1 and Table 2 and Figure 2).

Lastly, the Exon 14 CNV, found in 77% of participants in a heterozygous state and 6% in a homozygous state, was significantly associated with cognitive impairment (Table 1 and Table 2), further reinforcing the role of genetic mutations in objective cognitive decline, but the pre-defined significance level was not reached in the regression analysis (*p*-values are between 0.072 and 0.11 in the considered models) (Figure 2).

**Figure 3:**
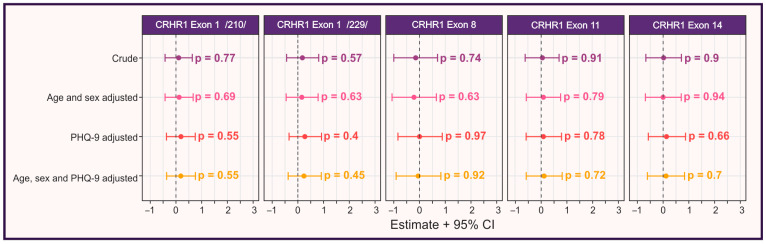
Association of self-reported cognitive impairment with *CRHR1* gene copy number variations in univariate analysis and after adjustment for depression (assessed by the Patient Health Questionnaire-9 [PHQ-9]), age, and sex. Error bars represent 95% confidence intervals.

Interestingly, while *CRHR1* gene CNVs were significantly associated with objective cognitive impairments, they did not appear to influence self-reported cognitive decline (see details below). No significant correlation was found between *CRHR1* gene Exon1/210/(*p* = 0.77), Exon 1/229/(*p* = 0.57), Exon 8 (*p* = 0.74), Exon 11 (*p* = 0.91), and Exon 14 (*p* = 0.9) CNVs and perceived cognitive issues, suggesting that self-reported cognitive impairment may not be directly influenced by genetic variations (Figure 3).

### 3.2. Analysis of Other Risk Factors for Cognitive Impairment Based on RBANS

We next assessed other potential risk factors for cognitive impairment; as measured by the RBANS. Age and sex did not show a significant association with cognitive decline (*p* = 0.076 for age; *p* = 0.10 for sex) based on these neurocognitive assessment results (Table 1). Age had a significant correlation with objective cognitive decline after adjustment with other risk factors (Figure 2). In contrast; we observed significant associations between HSV-1, HSV-2, and Hepatitis A infections and cognitive impairment. HSV-1 positivity was notably higher in the cognitively impaired group (*p* = 0.005); as were HSV-2 (*p* = 0.040) and Hepatitis A (*p* = 0.039); suggesting that these viral infections may contribute to cognitive decline, in individuals recovering from SARS-CoV-2 infection.

Other infections, including SARS-CoV-2 (*p* = 0.1), CMV (*p* > 0.9), HIV (*p* > 0.9), Epstein–Barr virus (*p* = 0.3), Hepatitis B (*p* = 0.2), and Hepatitis C (*p* = 0.5), showed no statistically significant association with cognitive impairment, although minor variations were observed across groups. Similarly, vitamin B12, vitamin D, and folate levels were not significantly associated with cognitive impairment in the study population (Table 2).

### 3.3. Analysis of Other Risk Factors for Cognitive Impairment Based on MOCA

A parallel analysis of cognitive impairment using the MOCA revealed that age was significantly associated with cognitive decline (*p* = 0.013), showing a trend toward older age in the impaired group (Table 2). However, sex did not significantly correlate with cognitive impairment (*p* = 0.9). Some viral infections were again found to be significant: HSV-1 (*p* = 0.005), HSV-2 (*p* = 0.038), and Hepatitis A (*p* = 0.019) were significantly associated with cognitive decline, reinforcing the role of viral infections in post-viral cognitive impairments.

Other infections, such as SARS-CoV-2 (*p* = 0.1), CMV (*p* = 0.6), HIV (*p* > 0.9), Epstein–Barr virus (*p* = 0.8), Hepatitis B (*p* = 0.7), and Hepatitis C (*p* = 0.5) did not show a statistically significant association with cognitive impairment. Also, nutritional factors such as vitamin B12, vitamin D, and folate were not significantly associated with cognitive decline, suggesting that these factors may not be as influential as initially hypothesized (Table 2).

### 3.4. Depression as a Potential Confounding Factor in Cognitive Decline

The role of depressive symptoms as a potential confounding factor in cognitive impairment was also explored. Depressive mood has no relation with objective cognitive decline (*p* > 0.45 for both considered models) (Table 3A). A significant association was observed between self-reported cognitive symptoms and questionnaire-based depression (*p* = 0.00078 for crude analysis and *p* = 0.0012 for the adjusted one (Table 3B). The estimate of about 0.12 (95% CI [0.05, 0.20]) for both models indicates that a one-point increase in PHQ score increases the chance of self-reported cognitive decline by approximately 1.12 times (95% CI [1.05, 1.22]). These results highlight depressive symptoms as a key factor in self-reported cognitive decline after SARS-CoV2 exposure.

We could argue that women demonstrate a higher susceptibility to depressive symptoms (*p* = 0.008). There is no statistically significant association between depressive mood and the other investigated risk factors, such as *CRHR1* gene Exon 1/210/, Exon 1/229/, Exon 8, Exon 11, and Exon 14 CNVs; hypovitaminosis B12, B9, and D; or different antecedent infections (SARS-CoV-2; HSV-1; HSV-2; CMV; EBV; Hepatitis A, B, and C; and HIV) (Table 4).

### 3.5. Self-Reported Cognitive Impairment and Its Association with Genetics, Depressive Symptoms, and Other Risk Factors

After assessing the association of several risk factors with objective cognitive impairment, we analyzed their effects on subjective (self-reported) cognitive decline. While some *CRHR1* CNVs were significantly associated with objective cognitive impairments, they did not show an association with self-reported cognitive decline (Exon 1/210, *p* = 0.77; Exon 1/229, *p* = 0.57; Exon 8, *p* = 0.74; Exon 11, *p* = 0.91; Exon 14, *p* = 0.9; Figure 2), suggesting that subjective cognitive impairment may not be directly influenced by *CRHR1* gene variations. Additionally, physiological factors (age, sex), vitamin deficiencies (B12, B9, D), and prior viral infections (SARS-CoV-2, HSV-1, HSV-2, CMV, EBV, hepatitis A–C, HIV) were not significantly associated with self-reported cognitive decline (Table 5, Figure 3). In contrast, depressive mood was strongly associated with subjective cognitive impairment (*p* = 0.00078; Table 3B and Table 4; Figure 2), indicating that self-reported cognitive symptoms are more likely influenced by depressive symptoms rather than by objective cognitive deficits or genetic variations.

## 4. Discussion

Cognitive impairment and depressed mood, which were frequent symptoms among patients in the acute and late phases of COVID-19, are serious indicators affecting the quality of life, especially among the younger population. There are several studies linking depression and cognitive impairment, but evidence of a causal relationship is unclear (including among survivors of COVID-19). Considering a variety of risk factors, the genetic basis of depression and cognitive impairment in COVID-19 survivors was also investigated, but mainly at the level of different SNPs [53].

*CRHR1* plays an important role in the hypothalamic–pituitary–adrenal (HPA) axis and contributes to behavioral and cognitive responses under stress. The HPA axis regulates the body’s response to stress, and dysregulation of this system has been implicated in stress-related cognitive impairment and mental disorders [54,55]. Alterations in the *CRHR1* gene, including copy number variations, may be associated with dysregulation of stress-response pathways, which could increase vulnerability to cognitive impairment and depressive symptoms through different biological mechanisms [35,56]. These mechanisms may involve CRH-mediated neuroinflammation, oxidative stress, and alterations in neuroplasticity, all of which have been implicated in psychiatric disorders [57,58]. In addition, *CRHR1* variants may influence neurotransmitter systems such as glutamatergic and dopaminergic signaling, which play key roles in mood regulation and emotional processing [59]. However, the directionality and functional consequences of these associations remain incompletely understood. Therefore, elucidating the links between *CRHR1* genetic alterations and cognitive impairment may be important for identifying vulnerable individuals and informing future preventive or therapeutic strategies, particularly in populations exposed to chronic stress or neuroinflammatory insults.

In our cohort, we identified several homozygous and heterozygous combinations of *CRHR1* gene variants. Notably, CNV mutations in Exon 1 (210) and Exon 11 were significantly associated with objective cognitive impairment among SARS-CoV-2-exposed patients. The CNVs identified in Exons 1 and 11 of the *CRHR1* gene correspond to regions critical for receptor function. Exon 1 encodes part of the extracellular N-terminal domain responsible for CRH binding, while Exon 11 contributes to the intracellular C-terminal domain that regulates G-protein coupling and receptor internalization [60]. Deletions affecting these exons may plausibly influence receptor structure or signaling [61]; however, the functional impact of partial exon deletions, particularly heterozygous deletions, remains uncertain and requires validation in dedicated functional studies.

Although these findings suggest that *CRHR1* variants may contribute to cognitive vulnerability following infection, the limited sample size of our study precludes drawing conclusions about their prevalence in the broader Armenian population or their definitive pathogenic role. Further translational studies are warranted to elucidate the mechanisms underlying this association. Given that *CRHR1* CNV mutations—particularly in Exon 1 (210) and Exon 8, which are also observed in our population—encode receptors central to stress responses, such alterations could modulate stress reactivity and predispose individuals to psychiatric or neurodegenerative conditions via neuroinflammatory mechanisms [62,63]. This raises an important research question: Can SARS-CoV-2, as a potent stressor, induce cognitive impairment in individuals harboring *CRHR1* gene variations or mutations?”. We also examined prior viral exposures in relation to cognitive outcomes. HSV-1, HSV-2, and Hepatitis A seropositivity were more frequent among participants with objective cognitive impairment. However, seropositivity reflects lifetime exposure rather than active or central nervous system infection, limiting biological inference. Moreover, because all participants had a documented history of SARS-CoV-2 infection, these findings do not allow determination of whether the observed associations are attributable to chronic viral exposure, SARS-CoV-2 infection itself, or their interaction. Future studies including SARS-CoV-2-negative control groups and virological markers of active infection are required to clarify these relationships.

In contrast, depression, gender, and vitamin deficiencies did not show significant correlations with objective cognitive decline.

One of the most notable findings of our study was the strong correlation between self-reported cognitive decline and depressive symptoms. In our study self-reported cognitive impairment was directly correlated with depressive mood, but no statistically significant association was identified with *CRHR1* gene CNVs or other risk variables.

This suggests that mood disturbances may influence how individuals perceive their cognitive abilities. Our results are consistent with growing evidence that subjective cognitive symptoms, often referred to as “brain fog,” may be more closely tied to emotional states like depression rather than actual cognitive dysfunction [7,8,9]. In contrast to objective measures of cognitive impairment, which did not correlate with depressive mood, self-reported cognitive decline was strongly influenced by the emotional state of the individuals. This underscores the need for clinicians to differentiate between subjective symptoms and objective cognitive impairment when evaluating patients recovering from COVID-19. A major strength of this study is the parallel assessment of objectively measured cognitive performance and subjective cognitive complaints. The observed dissociation between these two domains suggests that self-reported cognitive decline in post-COVID-19 populations may primarily reflect affective symptoms rather than true neurocognitive dysfunction. This distinction has important clinical implications, underscoring the need for objective cognitive testing when evaluating patients with persistent cognitive complaints following SARS-CoV-2 infection.

Antidepressant treatment, including selective serotonin reuptake inhibitors (SSRIs), may alleviate subjective cognitive complaints in patients with post-COVID-19 depression. While previous studies suggest interactions between stress-related genetic variants and antidepressant response [64,65], the potential influence of *CRHR1* CNVs on treatment outcomes in SARS-CoV-2–related cognitive or affective symptoms remains speculative and warrants further investigation.

Recent observations can also be interpreted in light of recent studies examining SARS-CoV-2 immune responses, including antibody dynamics against the spike (S) and nucleocapsid (N) proteins. Evidence indicates that the trajectories of S and N antibody levels differ across clinical phenotypes and may relate to long-term post-COVID-19 outcomes [66,67]. These immune signatures, although not directly linked to cognition or *CRHR1* in the existing literature, support the broader hypothesis that immune dysregulation interacts with genetic predisposition in shaping neuropsychiatric symptoms after COVID-19.

Our current findings can be contextualized within broader research on age-related immune responses, including the study by [68], which highlights how immune function changes with age may influence vulnerability to infection and inflammation. While that study does not address cognition or *CRHR1* directly, it reinforces the importance of considering immune dysregulation as a factor potentially interacting with genetic predispositions in post-COVID-19 neuropsychiatric symptoms.

These observations should be interpreted as contextual rather than mechanistic, as direct links between immune signatures, *CRHR1* variation, and cognitive outcomes were not assessed in the present study.

### 4.1. Limitations

This study has several limitations. First, although multiple covariates were examined, it was not possible to adjust for all potential confounders and effect modifiers of cognitive impairment. Second, the small sample size reflects the pilot nature of the study; all available and eligible subjects were included, but the findings should be interpreted with caution and considered preliminary. In addition, the measurements used were constrained by their respective levels of sensitivity and specificity.

A further limitation is that the study population consisted exclusively of individuals of Armenian nationality, which may limit generalizability to other ethnic groups. Genetic backgrounds and the prevalence of risk factors can vary across populations, potentially influencing the observed associations. Future studies should examine these associations in more diverse cohorts and ethnic groups to assess the universality of the findings and provide broader insights into genetic and environmental contributors to cognitive decline.

In addition, because all participants were SARS-CoV-2-positive, the study design allows evaluation of *CRHR1* CNVs as potential modifiers of post-COVID-19 cognitive impairment but does not permit conclusions regarding whether these CNVs are specifically related to SARS-CoV-2 infection. Inclusion of a SARS-CoV-2-negative control group would be necessary to determine infection-specific effects on *CRHR1* gene variation.

Finally, the cross-sectional design precludes causal inference, and information on pre-infection cognitive status was not available. The lack of functional assays also limits interpretation of the biological consequences of the identified *CRHR1* CNVs.

### 4.2. Implications for Clinical Practice and Future Research

Most cognitive impairments due to depression are reversible, but there are also persistent forms [69]. Interventions aimed at treating depression, such as SSRIs, may help alleviate these subjective symptoms among COVID-19 infected patients, improving the overall quality of life for patients suffering from both depressive symptoms and cognitive issues [25].

Future research is needed to clarify the pathogenicity of *CRHR1* genetic variations and underlying mechanisms of cognitive impairment in COVID-19 survivors and explore potential therapeutic strategies targeting both genetic and psychological factors.

## 5. Conclusions

This pilot study suggests that inter-individual variability in stress-response pathways, reflected by copy number variations in the *CRHR1* gene, may be associated with objectively measured cognitive impairment in adults following SARS-CoV-2 infection, alongside selected demographic and biological factors. In contrast, self-reported cognitive decline was primarily related to depressive symptoms rather than to objective neurocognitive performance.

The observed dissociation between subjective and objective cognitive outcomes represents an important strength of this study and highlights the need for objective cognitive assessment when evaluating persistent cognitive complaints in post-COVID-19 populations. Although the cross-sectional design and limited sample size preclude causal inference, these findings support the hypothesis that genetic susceptibility and affective state may differentially influence cognitive outcomes after viral infection. Larger, longitudinal studies are required to confirm these associations and to clarify their biological and clinical significance.

## Figures and Tables

**Table 1:** Descriptive statistics for risk factors based on cognitive impairment according to RBANS.

Variable	n	Overall n = 162 ^1^	High (≥90) n = 84 ^1^	Low (<90) n = 78 ^1^	*p*-Value ^2^
Age	162	41 (31, 52)	41 (30, 49)	42 (32, 56)	0.076
Sex	162				0.10
F		106 (65%)	50 (60%)	56 (72%)	
M		56 (35%)	34 (40%)	22 (28%)	
CMV IgG	125				>0.9
high		121 (97%)	64 (97%)	57 (97%)	
low		4 (3.2%)	2 (3.0%)	2 (3.4%)	
unknown		37	18	19	
HIVCOMPT	149				>0.9
high		1 (0.7%)	1 (1.3%)	0 (0%)	
low		148 (99%)	78 (99%)	70 (100%)	
unknown		13	5	8	
HSV-1	131				0.005
high		88 (67%)	43 (57%)	45 (80%)	
low		43 (33%)	32 (43%)	11 (20%)	
unknown		31	9	22	
HSV-2	129				0.040
high		9 (7.0%)	2 (2.7%)	7 (13%)	
low		120 (93%)	71 (97%)	49 (88%)	
unknown		33	11	22	
A-HCVII	142				0.5
high		1 (0.7%)	0 (0%)	1 (1.5%)	
low		141 (99%)	75 (100%)	66 (99%)	
unknown		20	9	11	
EBVEBNA IgG	134				0.3
high		120 (90%)	59 (87%)	61 (92%)	
low		14 (10%)	9 (13%)	5 (7.6%)	
unknown		28	16	12	
HBSAGII	148				0.2
high		5 (3.4%)	1 (1.3%)	4 (5.8%)	
low		143 (97%)	78 (99%)	65 (94%)	
unknown		14	5	9	
AHAV 2	129				0.039
high		22 (17%)	16 (24%)	6 (9.8%)	
low		107 (83%)	52 (76%)	55 (90%)	
unknown		33	16	17	
ACOV2 IgG	148				0.10
high		142 (96%)	77 (99%)	65 (93%)	
low		6 (4.1%)	1 (1.3%)	5 (7.1%)	
unknown		14	6	8	
B12 II	162				0.7
high		9 (5.6%)	4 (4.8%)	5 (6.4%)	
low		22 (14%)	10 (12%)	12 (15%)	
normal		131 (81%)	70 (83%)	61 (78%)	
VitD II	162				0.3
high		1 (0.6%)	1 (1.2%)	0 (0%)	
low		78 (48%)	37 (44%)	41 (53%)	
normal		83 (51%)	46 (55%)	37 (47%)	
FOL III	162				0.8
high		3 (1.9%)	2 (2.4%)	1 (1.3%)	
low		40 (25%)	22 (26%)	18 (23%)	
normal		119 (73%)	60 (71%)	59 (76%)	
*CRHR1* Exon 1/210/	151				0.008
0		63 (42%)	24 (31%)	39 (53%)	
1		78 (52%)	46 (59%)	32 (44%)	
2		10 (6.6%)	8 (10%)	2 (2.7%)	
unknown		11	6	5	
*CRHR1* Exon 1/229/	151				0.025
0		119 (79%)	58 (74%)	61 (84%)	
1		25 (17%)	13 (17%)	12 (16%)	
2		7 (4.6%)	7 (9.0%)	0 (0%)	
unknown		11	6	5	
*CRHR1* Exon 8	151				0.12
1		125 (83%)	61 (78%)	64 (88%)	
2		26 (17%)	17 (22%)	9 (12%)	
unknown		11	6	5	
*CRHR1* Exon 11	151				0.002
1		97 (64%)	41 (53%)	56 (77%)	
2		54 (36%)	37 (47%)	17 (23%)	
unknown		11	6	5	
*CRHR1* Exon 14	151				0.010
0		9 (6.0%)	7 (9.0%)	2 (2.7%)	
1		116 (77%)	52 (67%)	64 (88%)	
2		26 (17%)	19 (24%)	7 (9.6%)	
unknown		11	6	5	
MOCA TOTAL	161	26.0 (23.0, 28.0)	28.0 (26.0, 29.0)	24.0 (21.0, 26.0)	<0.001
unknown		1	0	1	
Memory impairment after COVID-19	162	92 (57%)	47 (56%)	45 (58%)	0.8

^1^ Median (Q1, Q3); n (%); ^2^ Wilcoxon rank sum test; Pearson’s Chi-squared test; Fisher’s exact test.

**Table 2:** Descriptive statistics for risk factors based on cognitive impairment according to MOCA.

Variable	n	Overall n = 161 ^1^	High (>25) n = 86 ^1^	Low (≤25) n = 75 ^1^	*p*-Value ^2^
Age	161	41 (31, 52)	38 (29, 51)	43 (36, 53)	0.013
Sex	161				0.9
F		106 (66%)	57 (66%)	49 (65%)	
M		55 (34%)	29 (34%)	26 (35%)	
CMV IgG	124				0.6
high		120 (97%)	65 (96%)	55 (98%)	
low		4 (3.2%)	3 (4.4%)	1 (1.8%)	
unknown		37	18	19	
HIVCOMPT	148				>0.9
high		1 (0.7%)	1 (1.3%)	0 (0%)	
low		147 (99%)	77 (99%)	70 (100%)	
unknown		13	8	5	
HSV-1	130				0.005
high		87 (67%)	42 (57%)	45 (80%)	
low		43 (33%)	32 (43%)	11 (20%)	
unknown		31	12	19	
HSV-2	128				0.038
high		9 (7.0%)	2 (2.7%)	7 (13%)	
low		119 (93%)	71 (97%)	48 (87%)	
unknown		33	13	20	
A-HCVII	141				0.5
high		1 (0.7%)	0 (0%)	1 (1.5%)	
low		140 (99%)	74 (100%)	66 (99%)	
unknown		20	12	8	
EBVEBNA IgG	133				0.8
high		119 (89%)	64 (89%)	55 (90%)	
low		14 (11%)	8 (11%)	6 (9.8%)	
unknown		28	14	14	
HBSAGII	147				0.7
high		5 (3.4%)	2 (2.6%)	3 (4.3%)	
low		142 (97%)	76 (97%)	66 (96%)	
unknown		14	8	6	
AHAV 2	128				0.019
high		22 (17%)	17 (24%)	5 (8.6%)	
low		106 (83%)	53 (76%)	53 (91%)	
unknown		33	16	17	
ACOV2 IgG	147				0.10
high		141 (96%)	78 (99%)	63 (93%)	
low		6 (4.1%)	1 (1.3%)	5 (7.4%)	
unknown		14	7	7	
B12 II	161				0.4
high		8 (5.0%)	6 (7.0%)	2 (2.7%)	
low		22 (14%)	10 (12%)	12 (16%)	
normal		131 (81%)	70 (81%)	61 (81%)	
VitD II	161				0.2
high		1 (0.6%)	0 (0%)	1 (1.3%)	
low		77 (48%)	37 (43%)	40 (53%)	
normal		83 (52%)	49 (57%)	34 (45%)	
FOL III	161				0.2
high		3 (1.9%)	0 (0%)	3 (4.0%)	
low		40 (25%)	21 (24%)	19 (25%)	
normal		118 (73%)	65 (76%)	53 (71%)	
*CRHR1* Exon 1/210/	150				0.016
0		63 (42%)	25 (32%)	38 (54%)	
1		78 (52%)	47 (59%)	31 (44%)	
2		9 (6.0%)	7 (8.9%)	2 (2.8%)	
unknown		11	7	4	
*CRHR1* Exon 1/229/	150				0.10
0		119 (79%)	58 (73%)	61 (86%)	
1		24 (16%)	15 (19%)	9 (13%)	
2		7 (4.7%)	6 (7.6%)	1 (1.4%)	
unknown		11	7	4	
*CRHR1* Exon 8	150				0.003
1		125 (83%)	59 (75%)	66 (93%)	
2		25 (17%)	20 (25%)	5 (7.0%)	
unknown		11	7	4	
*CRHR1* Exon 11	150				0.002
1		97 (65%)	42 (53%)	55 (77%)	
2		53 (35%)	37 (47%)	16 (23%)	
unknown		11	7	4	
*CRHR1* Exon 14	150				0.022
0		9 (6.0%)	3 (3.8%)	6 (8.5%)	
1		116 (77%)	57 (72%)	59 (83%)	
2		25 (17%)	19 (24%)	6 (8.5%)	
unknown		11	7	4	
RBANS Total Scale of Index Scores	161	90 (82, 100)	98 (89, 105)	84 (78, 90)	<0.001
Memory impairment after COVID-19	161	92 (57%)	52 (60%)	40 (53%)	0.4

^1^ Median (Q1, Q3); n (%); ^2^ Wilcoxon rank sum test; Pearson’s Chi-squared test; Fisher’s exact test.

**Table 3:** Impact of depressive mood. (**A**). Impact of depressive mood on objective cognitive decline. (**B**). Impact of depressive mood on self-reported cognitive decline.

(**A**)							
Type	term	estimate	std.error	statistic	*p*.value	conf.low	conf.high
Impact of PHQ-9	‘PHQ-9’	−0.02810	0.0497	−0.5650	0.573000	−0.125	0.0692
Age/sex adjustment	‘PHQ-9’	−0.03710	0.0490	−0.7560	0.451000	−0.133	0.0590
	Age	−0.07740	0.0207	−3.7400	0.000255	−0.118	−0.0369
	SexM	−0.00608	0.5620	−0.0108	0.991000	−1.110	1.0900
(**B**)							
Type	term	estimate	std.error	statistic	*p*.value	conf.low	conf.high
Impact of PHQ-9	‘PHQ-9’	0.1220	0.0364	3.360	0.000781	0.0545	0.1980
Age/sex adjustment	‘PHQ-9’	0.1190	0.0370	3.230	0.001240	0.0505	0.1960
	Age	0.0117	0.0136	0.858	0.391000	−0.0149	0.0387
	SexM	−0.2120	0.3610	−0.588	0.557000	−0.9190	0.5000

**Table 4:** Association of risk factors with depressive symptoms.

Variable	n	Overall n = 161 ^1^	High (≥5) n = 99 ^1^	Low (<5) n = 62 ^1^	*p*-Value ^2^
Age	161	41 (31, 52)	42 (31, 53)	41 (31, 51)	0.7
Sex	161				0.008
F		106 (66%)	73 (74%)	33 (53%)	
M		55 (34%)	26 (26%)	29 (47%)	
CMV IgG	124				0.2
high		120 (97%)	72 (95%)	48 (100%)	
low		4 (3.2%)	4 (5.3%)	0 (0%)	
unknown		37	23	14	
HIVCOMPT	148				>0.9
high		1 (0.7%)	1 (1.1%)	0 (0%)	
low		147 (99%)	88 (99%)	59 (100%)	
unknown		13	10	3	
HSV-1	130				0.6
high		87 (67%)	50 (65%)	37 (70%)	
low		43 (33%)	27 (35%)	16 (30%)	
unknown		31	22	9	
HSV-2	128				0.7
high		9 (7.0%)	6 (7.9%)	3 (5.8%)	
low		119 (93%)	70 (92%)	49 (94%)	
unknown		33	23	10	
A-HCVII	141				>0.9
high		1 (0.7%)	1 (1.1%)	0 (0%)	
low		140 (99%)	86 (99%)	54 (100%)	
unknown		20	12	8	
EBVEBNA IgG	133				0.8
high		119 (89%)	72 (89%)	47 (90%)	
low		14 (11%)	9 (11%)	5 (9.6%)	
unknown		28	18	10	
HBSAGII	147				0.4
high		5 (3.4%)	2 (2.2%)	3 (5.2%)	
low		142 (97%)	87 (98%)	55 (95%)	
unknown		14	10	4	
AHAV 2	128				0.7
high		22 (17%)	14 (18%)	8 (15%)	
low		106 (83%)	62 (82%)	44 (85%)	
unknown		33	23	10	
ACOV2 IgG	147				>0.9
high		141 (96%)	86 (96%)	55 (96%)	
low		6 (4.1%)	4 (4.4%)	2 (3.5%)	
unknown		14	9	5	
B12 II	161				0.2
high		8 (5.0%)	7 (7.1%)	1 (1.6%)	
low		22 (14%)	11 (11%)	11 (18%)	
normal		131 (81%)	81 (82%)	50 (81%)	
VitD II	161				0.8
high		1 (0.6%)	1 (1.0%)	0 (0%)	
low		77 (48%)	46 (46%)	31 (50%)	
normal		83 (52%)	52 (53%)	31 (50%)	
FOL III	161				0.9
high		3 (1.9%)	2 (2.0%)	1 (1.6%)	
low		40 (25%)	26 (26%)	14 (23%)	
normal		118 (73%)	71 (72%)	47 (76%)	
*CRHR1* Exon 1/210/	150				0.8
0		63 (42%)	38 (40%)	25 (45%)	
1		78 (52%)	51 (54%)	27 (48%)	
2		9 (6.0%)	5 (5.3%)	4 (7.1%)	
unknown		11	5	6	
*CRHR1* Exon 1/229/	150				0.5
0		119 (79%)	75 (80%)	44 (79%)	
1		24 (16%)	16 (17%)	8 (14%)	
2		7 (4.7%)	3 (3.2%)	4 (7.1%)	
unknown		11	5	6	
*CRHR1* Exon 8	150				0.8
1		125 (83%)	79 (84%)	46 (82%)	
2		25 (17%)	15 (16%)	10 (18%)	
unknown		11	5	6	
*CRHR1* Exon 11	150				0.4
1		97 (65%)	63 (67%)	34 (61%)	
2		53 (35%)	31 (33%)	22 (39%)	
unknown		11	5	6	
*CRHR1* Exon 14	150				0.6
0		9 (6.0%)	5 (5.3%)	4 (7.1%)	
1		116 (77%)	75 (80%)	41 (73%)	
2		25 (17%)	14 (15%)	11 (20%)	
unknown		11	5	6	
MOCA TOTAL	161	26.0 (23.0, 28.0)	26.0 (23.0, 29.0)	25.5 (24.0, 28.0)	0.8
RBANS Total Scale of Index Scores	161	90 (82, 100)	90 (79, 99)	91 (83, 102)	0.3
Memory impairment after COVID-19	161	92 (57%)	66 (67%)	26 (42%)	0.002

^1^ Median (Q1, Q3); n (%); ^2^ Wilcoxon rank sum test; Pearson’s Chi-squared test; Fisher’s exact test.

**Table 5:** Descriptive statistics of self-reported cognitive impairment after COVID-19 with possible risk factors.

Variable	n	Overall n = 162 ^1^	0 n = 70 ^1^	1 n = 92 ^1^	*p*-Value ^2^
Age	162	41 (31, 52)	39 (32, 54)	43 (31, 52)	0.5
Sex	162				0.11
F		106 (65%)	41 (59%)	65 (71%)	
M		56 (35%)	29 (41%)	27 (29%)	
CMV IgG	125				0.6
high		121 (97%)	54 (98%)	67 (96%)	
low		4 (3.2%)	1 (1.8%)	3 (4.3%)	
unknown		37	15	22	
HIVCOMPT	149				>0.9
high		1 (0.7%)	0 (0%)	1 (1.2%)	
low		148 (99%)	66 (100%)	82 (99%)	
unknown		13	4	9	
HSV-1	131				0.3
high		88 (67%)	42 (72%)	46 (63%)	
low		43 (33%)	16 (28%)	27 (37%)	
unknown		31	12	19	
HSV-2	129				>0.9
high		9 (7.0%)	4 (7.0%)	5 (6.9%)	
low		120 (93%)	53 (93%)	67 (93%)	
unknown		33	13	20	
A-HCVII	142				>0.9
high		1 (0.7%)	0 (0%)	1 (1.3%)	
low		141 (99%)	62 (100%)	79 (99%)	
unknown		20	8	12	
EBVEBNA IgG	134				0.3
high		120 (90%)	50 (86%)	70 (92%)	
low		14 (10%)	8 (14%)	6 (7.9%)	
unknown		28	12	16	
HBSAGII	148				0.7
high		5 (3.4%)	3 (4.5%)	2 (2.4%)	
low		143 (97%)	63 (95%)	80 (98%)	
unknown		14	4	10	
AHAV 2	129				0.2
high		22 (17%)	13 (22%)	9 (13%)	
low		107 (83%)	46 (78%)	61 (87%)	
unknown		33	11	22	
ACOV2 IgG	148				>0.9
high		142 (96%)	61 (95%)	81 (96%)	
low		6 (4.1%)	3 (4.7%)	3 (3.6%)	
unknown		14	6	8	
B12 II	162				0.4
high		9 (5.6%)	5 (7.1%)	4 (4.3%)	
low		22 (14%)	12 (17%)	10 (11%)	
normal		131 (81%)	53 (76%)	78 (85%)	
VitD II	162				0.3
high		1 (0.6%)	0 (0%)	1 (1.1%)	
low		78 (48%)	38 (54%)	40 (43%)	
normal		83 (51%)	32 (46%)	51 (55%)	
FOL III	162				0.2
high		3 (1.9%)	0 (0%)	3 (3.3%)	
low		40 (25%)	15 (21%)	25 (27%)	
normal		119 (73%)	55 (79%)	64 (70%)	
*CRHR1* Exon 1/210/	151				0.13
0		63 (42%)	30 (48%)	33 (38%)	
1		78 (52%)	27 (43%)	51 (58%)	
2		10 (6.6%)	6 (9.5%)	4 (4.5%)	
unknown		11	7	4	
*CRHR1* Exon 1/229/	151				0.9
0		119 (79%)	50 (79%)	69 (78%)	
1		25 (17%)	11 (17%)	14 (16%)	
2		7 (4.6%)	2 (3.2%)	5 (5.7%)	
unknown		11	7	4	
*CRHR1* Exon 8	151				0.6
1		125 (83%)	51 (81%)	74 (84%)	
2		26 (17%)	12 (19%)	14 (16%)	
unknown		11	7	4	
*CRHR1* Exon 11	151				0.9
1		97 (64%)	40 (63%)	57 (65%)	
2		54 (36%)	23 (37%)	31 (35%)	
unknown		11	7	4	
*CRHR1* Exon 14	151				0.6
0		9 (6.0%)	5 (7.9%)	4 (4.5%)	
1		116 (77%)	46 (73%)	70 (80%)	
2		26 (17%)	12 (19%)	14 (16%)	
unknown		11	7	4	
MOCA TOTAL	161	26.0 (23.0, 28.0)	25.0 (23.0, 28.0)	26.0 (24.0, 28.0)	0.5
unknown		1	1	0	
RBANS Total Scale of Index Scores	162	90 (81, 100)	91 (81, 100)	90 (82, 99)	0.8

^1^ Median (Q1, Q3); n (%); ^2^ Wilcoxon rank sum test; Pearson’s Chi-squared test; Fisher’s exact test.

## Data Availability

The data presented in this study are only available on request from the corresponding author due to ethical restrictions.
